# Development of a Printed Sensor and Wireless Measurement System for Urination Monitoring

**DOI:** 10.3390/s25102961

**Published:** 2025-05-08

**Authors:** Lan Zhang, En Takashi, Jian Lu, Sohei Matsumoto

**Affiliations:** 1 Device Technology Research Institute, National Institute of Advanced Industrial Science and Technology (AIST), Tsukuba, Ibaraki 305-8564, Japan; jian-lu@aist.go.jp (J.L.); sohei.matsumoto@aist.go.jp (S.M.); 2 Faculty of Nursing, Nagano College of Nursing, Akaho, Komagane, Nagano 399-4117, Japan; takashi@nagano-nurs.ac.jp

**Keywords:** urinary monitoring sensor, animal health monitoring, real-time measurement, sensor printing technology

## Abstract

The development of reliable and efficient sensors is essential for advances in health monitoring technologies. This study focused on the fabrication and evaluation of a multichannel printed sensor electrode designed for long-term stability and effective data acquisition. Using rapid printing technology, we created a urine sensor array with extended electrodes for the measurement of urine volume and frequency. The ultrathin design of the sensor electrode, with an average thickness of only 30 microns, ensures both user comfort and measurement accuracy. The sensor electrode dimensions were meticulously designed, measured, and optimized through successful trial manufacturing of the sensor electrode and sensor array. Comprehensive evaluation of the fabricated sensor demonstrated excellent performance, including a high response speed of ≤1 s and long-term stability exceeding 5 weeks. In addition, wireless transmission capabilities and user interfaces were developed for field experiments. Finally, animal experiments were performed to evaluate the field performance of the fabricated sensor. Accordingly, we are confident that the sensor developed herein will contribute to enhancing healthcare in an aging society.

## 1. Introduction

With continuous modernization and improvements in living standards, healthcare has become a major focus within society [1,2]. At present, societies worldwide face the challenges of population decline and aging, which result in an increasing prevalence of age-associated health issues [3,4]. This social phenomenon is accompanied by a shortage of the necessary medical resources. When people are not satisfied with their health, they usually make lifestyle adaptations, such as increasing physical exercise and eating a healthy diet; however, the use of pharmaceuticals remains among the most effective interventions [5,6]. In the field of preclinical drug development, rats are among the most common model organisms used to test the efficacy and safety of newly developed compounds [7,8,9]. By measuring various physiological parameters of animals after drug intake, researchers gain insight into drug responses. At present, rats are extensively used in the development of drugs against cardiovascular diseases, cancer, and neurological diseases, as well as in vaccine development. Monitoring urine excretion in real time during drug evaluation can provide researchers with a wealth of valuable information, such as the health status of rats and drug metabolism [10,11]. Although there are multiple smart and useful cage systems that can monitor the physiological characteristics of rats in real time, these are relatively expensive and difficult to operate [12,13]. Further, in some cases, rats are transferred from a familiar living environment to an unfamiliar observation box, which requires a period of adaptation, thus compromising time efficiency.

In this study, we developed a new urine monitoring system with an ultrathin sensor electrode array that can easily be set under a rat cage without any impact on the living state of target animals. To validate the performance of the sensor electrode, we conducted a series of experiments focusing on its electrical characteristics and practical application in urination monitoring. The key parameters evaluated included sensitivity, response time, and stability over prolonged usage. Sensitivity tests were conducted by applying known quantities of liquid to the sensor surface and measuring the corresponding electrical output. The response time was assessed by monitoring the time required for the sensor to detect changes in its environment and relay this information to the transmission unit. Stability tests involved continuous sensor operation for several hours at intervals of several weeks to record drift or degradation. We then evaluated our sensor system’s ability to assess urination time, volume, and patterns in rats. The measurement results were analyzed to characterize the individual urine profiles of rats. This approach is not limited to the study of urination behavior in rats, and can also be applied to other research areas and drug testing. In the future, we intend to expand the use of this technology to veterinary diagnostics [14,15].

**Figure 1:**
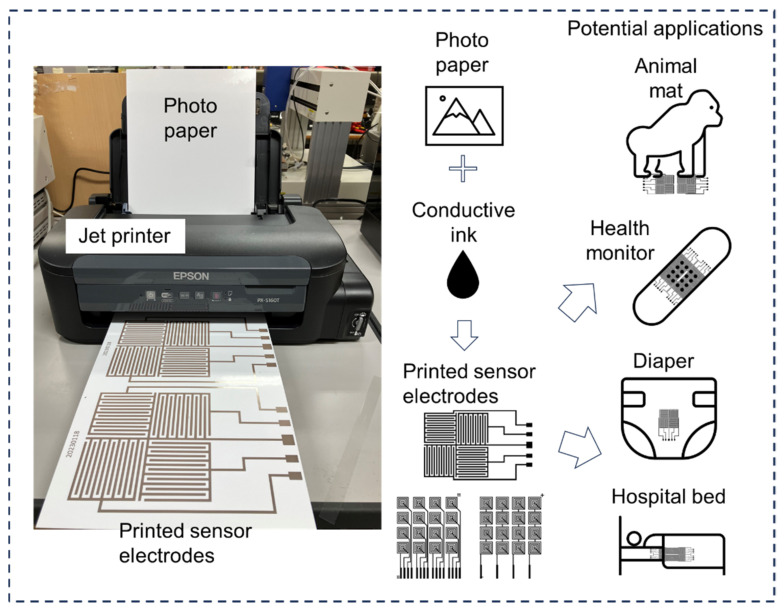
Schematic illustration of the fabrication process and potential applications of the sensor array for urinary monitoring.

We previously developed a rapid manufacturing platform for fabricating urine sensors [16], which enables the production of flexible and thin urine detection sensors on uneven surfaces through noncontact injection technology. However, the complexity and high cost of this process restricts its large-scale application. In recent years, numerous researchers have developed highly efficient and user-friendly urine testing systems [17,18,19,20]. However, these systems often require relatively expensive fabrication processes or complex electrode preparation, which partly limits their large-scale application. The sensor electrode manufacturing process described in this study adopted the direct printing electrode method, which is highly suitable for relatively flat surfaces and significantly reduces costs. Figure 1 demonstrates the fabrication platform of the urine sensor electrode, along with the design concept and potential applications of the developed sensor system. The materials and equipment used for sensor electrode manufacturing are readily accessible, and the initial investment cost is relatively low. A standard monochrome inkjet printer was used to fabricate the sensor electrodes directly onto the substrates. Conductive ink was loaded into the printer’s tank for electrode production, and photopaper served as the printing substrate. The resulting sensor electrodes achieved a thickness of only tens of microns, ensuring minimal impact on user comfort. A urine sensor can be placed on a diaper or mat to continuously monitor urine volume status without affecting the user’s normal life. Owing to its low cost, the manufactured ultrathin urine sensor can be adapted for various applications. For example, it can be used to measure the duration and volume of urination in infants or older people in real time. Urine volume detection is essential to healthcare in the older population, contributing to disease prevention. Future work will focus on expanding the functionality of the urine sensor system by changing the electrode material and measurement circuit to detect urine contents, such as various ions, proteins, urine occult blood, and other biological indicators.

## 2. Fabrication of the Sensor Array with a Measurement System

**Figure 2:**
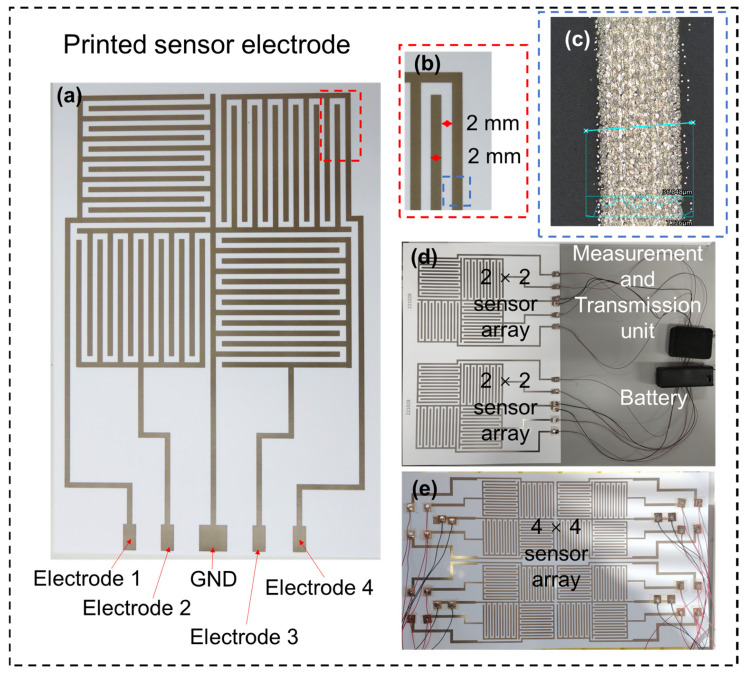
Fabricated typical urinary monitoring sensor array. (**a**,**b**) Optical image of a 2 × 2 sensor array and the comb electrode. (**c**) Enlarged view of the electrode beam. (**d**) Two sets of 2 × 2 sensor arrays with measurement and transmission units for field animal measurements. (**e**) Optical image of a typical 4 × 4 sensor array.

The sensor fabrication process involves facilitative printing techniques that allow the creation of fine and precise patterns on flexible substrates. This flexibility is crucial to ensure that the sensor electrodes can be comfortably integrated into various mediating and nursing supplies. Figure 2 shows a typical urinary monitoring sensor array. The sensor unit was designed and fabricated with a classic full-bridge configuration, providing precise dimensional adjustability and eliminating blind spots in the testing area. The conductive AgIC ink is directly printed onto photographic paper to form a single-layer sensor electrode, without the need for a post-drying process. To ensure good conductivity of the electrode lines, the jet printer’s gamma value was set to 2.2, brightness was set to −25, contrast was set to 25, and the print quality was set to high precision. The reason for choosing conductive AgIC ink is its low internal resistance and excellent ductility. Sensors printed on photographic paper and special plastics exhibit elongation rates exceeding 5% and 20%, respectively, making them well suited for applications involving large deformations. We had previously experimented with carbon-based inks, which offer the advantage of lower cost. However, their low ductility significantly limits their usability. Nevertheless, in certain special cases—such as large-area testing—carbon-based inks can still offer relative advantages. Figure 2a,b show an optical image of the fabricated 2 × 2 sensor array and the comb electrode structure. Figure 2c shows an enlarged view of the electrode beam with an average measured thickness of 30 μm. The sensor electrode was fabricated using a printing method to ensure precision and uniformity. The electrode array consisted of multiple individual electrodes, each measuring 2 mm in width, with a total span of 50 mm. The array configuration included four distinct electrodes (electrodes 1, 2, 3, and 4), along with a ground electrode. This arrangement was optimized to capture the detailed and accurate measurements necessary for the intended application. The sensor system included measurement and transmission units. The electrodes are connected to a circuit board using an extended wire. Each electrode in the array is connected to a measurement and transmission unit, which is responsible for collecting the electrical signals generated by the sensor (Figure 2d,e). These signals are then wirelessly transmitted to a central processing unit for further analysis. The user can observe the status of the terminal unit sensor in real time using a Bluetooth signal.

## 3. Fabrication and Evaluation Methods

Conductive AgIC ink (#1000, Elephantech Inc., Tokyo, Japan) was used to print the urine sensor electrodes. A monochrome inkjet printer (PX-S160T, Seiko Epson Co., Tokyo, Japan) was used to fabricate sensor electrodes on the target substrate. Photo paper (KA420SLU, Seiko Epson Co., Tokyo, Japan) was used as a substrate for printing the electrodes. A resistivity processor (sigma-5+, NPS Inc., Tokyo, Japan) was used to measure the sheet resistance of the sensor electrode beam. This device uses a four-point probe method to measure the sheet resistance of thin layers. A 3D printer (Ultimaker 2+, Ultimaker Co., Ltd., Utrecht, The Netherlands) was used to fabricate the packaged sensors and bracket structures. A 3D laser scanning microscope (VK-X3000, Keyence Co., Ltd., Osaka, Japan) was used to measure the dimensions and topography of the fabricated sensor electrode. A low-noise power supply unit (U8002A, Keysight Technologies Co., Ltd., Santa Clara, CA, USA) was used to provide power to the sensor electrodes, and a data logger (DL, Hv gl2000, Graphtec Co., Ltd., Kanagawa, Japan) was used to record the results. A pressure-sensitive adhesive (electrically conductive adhesive transfer tape 9703; 3M Co., Ltd., Maplewood, MN, USA) was used to connect the printed electrode array and wire-to-sensor system.

Two eight-week-old healthy male hairless rats (HWY/Slc; Japan SLC, Inc., Hamamatsu, Japan) were used in this study. Rats were placed in a standard cage (with a dimension size of 240 × 160 × 160 mm). A sheet mat covered the sensor array used to collect urine, and the highly absorbent polymer used was capable of absorbing and retaining moisture. The rats were fed a standard chow diet with ad libitum access to water and maintained on a 12 h light/dark cycle throughout the experiment. They were not handled or disturbed during the study. For the field measurements, the mat was designed to fit the rat cage and trimmed to a size of 240 × 160 mm. The field measurements were performed for 6 h. The data-sampling interval was set to 1 min. In addition, the ambient temperature in the animal room was 25 °C, and the relative humidity was 50%. This study was performed in strict accordance with the Guide for the Care and Use of Laboratory Animals of the National Institutes of Health. The protocol was approved by the Committee on the Ethics of Animal Experiments of Nagano College of Nursing (protocol code: No.2021-7).

## 4. Evaluation of the Fabricated Sensor Array

### 4.1. Sheet Resistance Measurement of the Fabricated Electrode Beam

**Figure 3:**
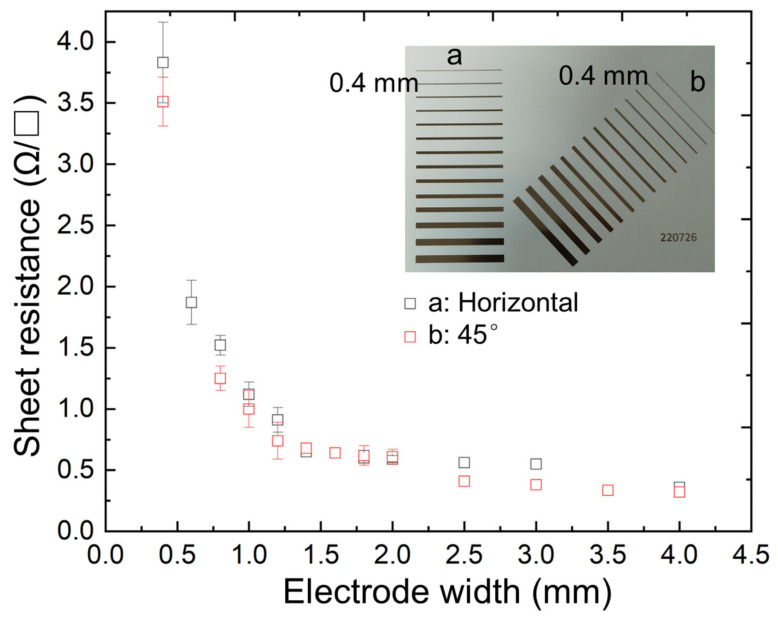
Comparison of the measured sheet resistance and the electrode beam width.

Figure 3 shows the results of the sheet resistance measurements of the fabricated electrode beams. The optical image in the inset of Figure 3 shows the two types of electrode beams that were tested: horizontal and 45-degree. The uniformity of the electrodes was confirmed by testing both types. All tested beams had a length of 50 mm and a width ranging from 0.2 to 4 mm. The measurement results revealed that as the width of the electrode beam increased, the sheet resistance decreased. This phenomenon indicates that a wider electrode beam has a lower sheet resistance. We observed that when the electrode beam width was 0.4 mm, the sheet resistances of both the horizontal and 45-degree printed electrodes were greater than 3.4 Ω/□. However, when the electrode width increased to 1.2 mm, the sheet resistance was markedly reduced to an average of 0.8 Ω/□. Based on the experimental results, we obtained design guidelines for the sensor electrode. In future fabrication processes, the width of the sensor electrode should be controlled to be wider than 1.2 mm to maintain the internal resistance of the sensor within an acceptable range. However, to minimize the sensor area, the electrode width can be adjusted to become narrower, which may complicate detection.

### 4.2. Evaluation of Full Response and Recovery Rate of the Sensor Electrode

**Figure 4:**
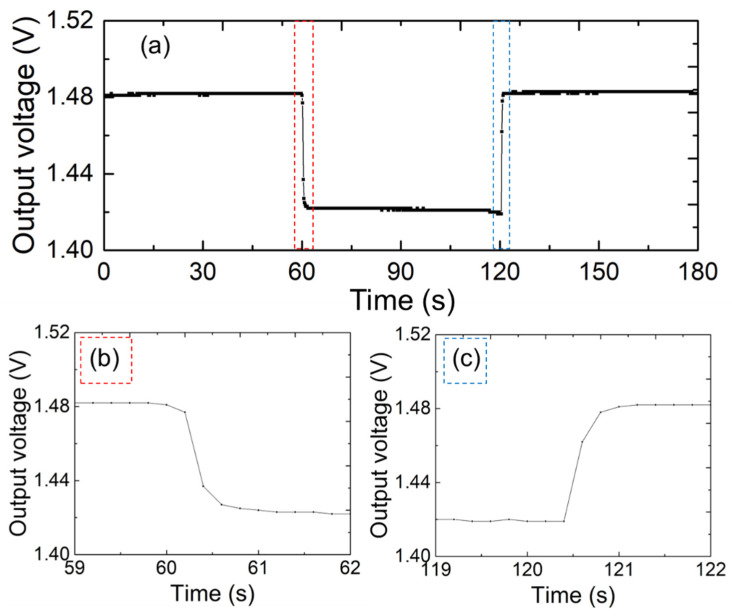
(**a**) Response speed of the given sensor under the mats with different moisture levels. (**b**) Response time and (**c**) recovery time of the sensor.

To verify the real-time monitoring capability of the sensor electrodes, we conducted comprehensive measurements of the sensor response and recovery time. Figure 4 shows the full response and recovery curves of the sensor under different moisture levels when placed on a mat. Figure 4b,c show the response and recovery times of the fabricated sensors, respectively. Two mats of the same size of 50 × 50 mm^2^ were alternately placed on top of the sensor electrodes for this experiment. One mat was in its original dry state, while the other had 0.5 mL of pure water diffused into it. As shown in Figure 4b,c, the sensor had a response time of 1 s and a recovery time of sub-seconds when the mat was filled with the solution. These results indicate that the proposed sensor responds to moisture changes with a speed comparable to that of well-established sensors [21]. The results of our evaluation indicated that the printed sensor electrode exhibits good sensitivity and quick response times, making it highly suitable for real-time health monitoring applications.

### 4.3. Interval Measurement Using One Sensor Electrode

**Figure 5:**
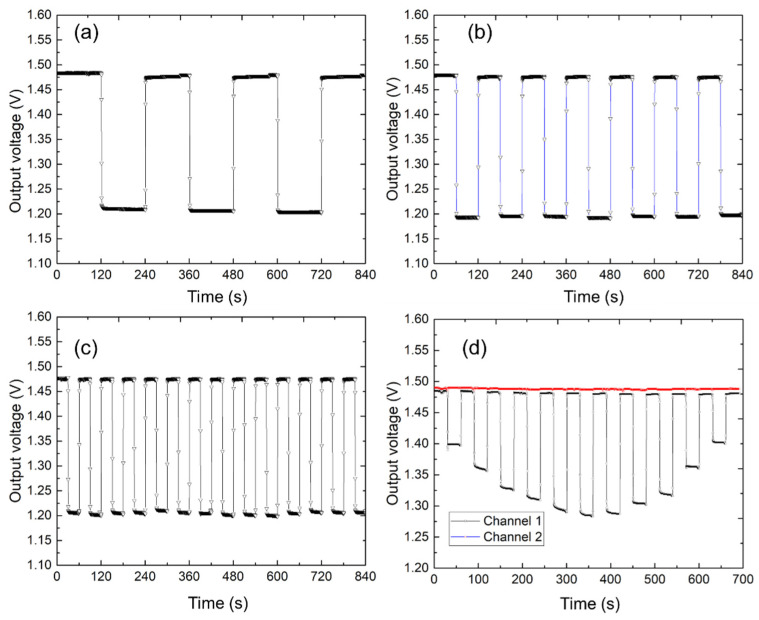
Interval measurement of the sensor electrode. (**a**–**c**) Sensor output under a mat dipped with pure water at 120, 60, and 30 s intervals. (**d**) Sensor output under a mat dipped with 0.5 to 3 mL of pure water at 0.5 mL intervals.

Interval measurements were conducted using the electrode units in a multi-channel sensor array. This measurement was undertaken to verify the precise monitoring of moisture change events by recording the electrical signals generated by the sensor upon the detection of urine. Figure 5 shows the interval measurement results for the sensor electrode. The electrode units in a multi-channel sensor array were used in this experiment, and the single sensing area of the electrode unit was 50 × 50 mm^2^. Figure 5a–c show the sensor output when a 50 × 50 mm^2^ mat, soaked with 5 mL of pure water, was placed on the electrode unit at 120, 60, and 30 s intervals. Figure 5d shows the measurements of the mat dipped with 0.5 to 3 mL of pure water at 0.5 mL intervals and placed on the electrode unit at 60 s intervals. As indicated by the results, the given sensor electrode could accurately measure the moisture change at these intervals. The sensor system collected continuous data and captured the exact timing and duration of moisture change events. Each detected moisture change event was represented as a distinct peak in the recorded signal, allowing for the temporal analysis of urination patterns. The amplitude and frequency of peaks in the sensor output corresponded to the volume and frequency of the moisture change events, respectively. By analyzing these peaks, we determined the intervals between moisture change events and the consistency of the urination patterns over time. Moreover, Figure 5d provides a reference for calibrating the sensor system. By calculating the moisture content and the output voltage, the sensor’s responsivity can be obtained. However, due to differences in the rate of moisture diffusion across different mats, the sensor does not produce a linear output. Therefore, individual calibration is required for each type of mat in order to achieve accurate quantitative measurements.

### 4.4. Long-Term Measurement with the Urine Sensor

**Figure 6:**
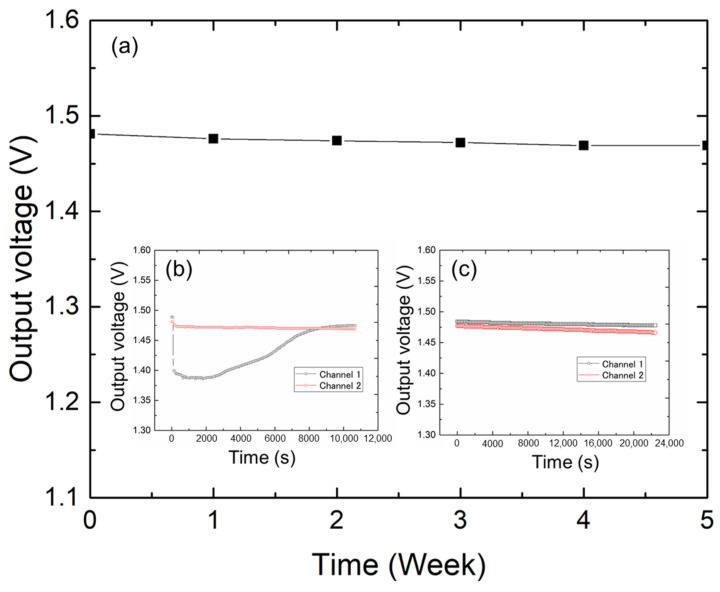
Long-term measurement of the proposed sensor electrodes. (**a**) Sensor output characteristics measured every other week. (**b**) Two identical sets of sensors and mats were tested by dropping 0.5 mL of liquid on one mat at 1 s intervals for a total duration of 11,000 s. (**c**) Two identical sets of sensors and mats were continuously tested for a total duration of 22,000 s.

Long-term stability is a crucial factor in evaluating the operational quality of sensors. The long-term measurement of urine sensors requires the consideration of two key reference indicators: (1) the stability of the sensor from the time it is printed on the substrate to when it is put into use; (2) the long-term stability of the sensor during continuous usage. We assessed the long-term stability of the sensor in terms of placement and continuous operation. To this end, we prepared a typical sensor with the dimensions of 50 × 50 mm^2^. To simulate a real use environment, we placed an absorbent mat above the measured sensor. Figure 6 shows the long-term results. First, the sensor output characteristics were tested every other week. Based on these results, the sensor maintained a consistent bottom line from the beginning of the test to the fifth week. The slight change in output can be attributed to moisture in the sensor base or mat. In this study, the long-term stabilities of the two sensors were compared. As shown in Figure 6b, we prepared two sets of identical sensors and mats and dropped 0.5 mL of liquid on one mat for 11,000 s of contact testing at 1 s intervals. The moisture in the mat in channel 1 evaporated over time, and the sensor’s output eventually returned to its original value. Figure 6c shows two identical sets of sensors and mats continuously tested for a total duration of 22,000 s. The sensor demonstrated a consistent performance with minimal signal drift over extended periods, confirming its long-term stability. This experiment was conducted in a laboratory with ambient room temperature and humidity. Therefore, the sensor substrate may absorb moisture from the air during long-term use, potentially leading to aging effects and slight signal drifts (Figure 6). These observations also provide important guidelines for sensor storage: the fabricated sensors should be stored in an environment with stable temperature and as low humidity as possible.

## 5. Field Measurement Using the Sensor System

### 5.1. Wireless Measurement, Transmission System, and User Interface

**Figure 7:**
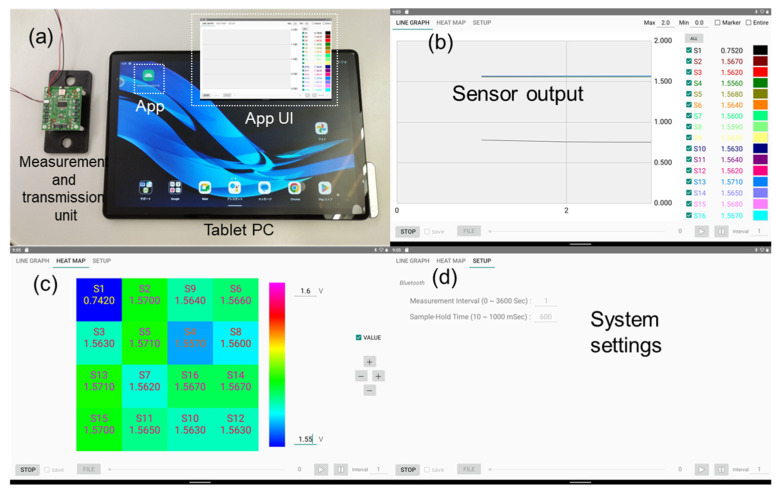
Wireless measurement and transmission system and user interface. (**a**) Optical image of the sensor system. (**b**–**d**) Monitoring, operation, and setting interfaces of the sensor system.

Figure 7 shows the wireless measurement and transmission system along with the user interface for the urinary monitoring sensor system. This system was designed to facilitate real-time data acquisition, transmission, and analysis, thereby providing a comprehensive solution for urination monitoring. Figure 7a shows an optical photograph of the urinary monitoring sensor system, primarily composed of hardware, software, and firmware components. The measurement and transmission circuits were integrated into a 40 mm × 34 mm compact printed circuit board. Sixteen-channel sockets were positioned on the front and back of the measurement and transmission unit boards to enable connection with the sensor electrodes. The system is designed to be compact, portable, and suitable for use in various monitoring environments. We selected and integrated a Bluetooth unit with multiple I/O interfaces for wireless communication. Using the SEGGER Embedded Studio 5.60 integrated development environment, an Android-based application was coded using the C and Kotlin programs. Regarding the power supply, two options were available: a stable 3.6 V battery supply or a 1.5–6 V supply using a DC-DC voltage converter. A tablet PC (LAVIE Tab T10, NEC Co., Tokyo, Japan) was used as the terminal device for displaying the measured data. Since the subjects of this empirical experiment were rats, and the breeding room was no larger than 10 m^2^, the Bluetooth transmission range integrated into this sensor system was sufficient to meet the experimental requirements. Wireless transmission tests confirmed that the tablet terminal could stably receive sensor signals within a 10 m range, with a packet loss rate of less than 5%. Additionally, the system recorded data with timestamps, ensuring the reliability and traceability of sensor data collection.

Figure 7b,c show the sensor output data with line and mapping graphs, respectively. This interface allows users to monitor ongoing urination events and track sensor performance. Users can select an appropriate display option based on the specific application requirements. Figure 7d shows the application setting page, which provides a user-friendly platform for interaction with the sensor system. The wireless measurement and transmission system, combined with a user-friendly interface, offers an effective solution for the real-time urination monitoring of animals. The system design ensured accurate data collection, reliable transmission, and easy access to data analysis tools, making it a valuable asset for animal healthcare and research applications. The ability to adjust the system settings and monitor data remotely further enhances its versatility and effectiveness.

**Figure 8:**
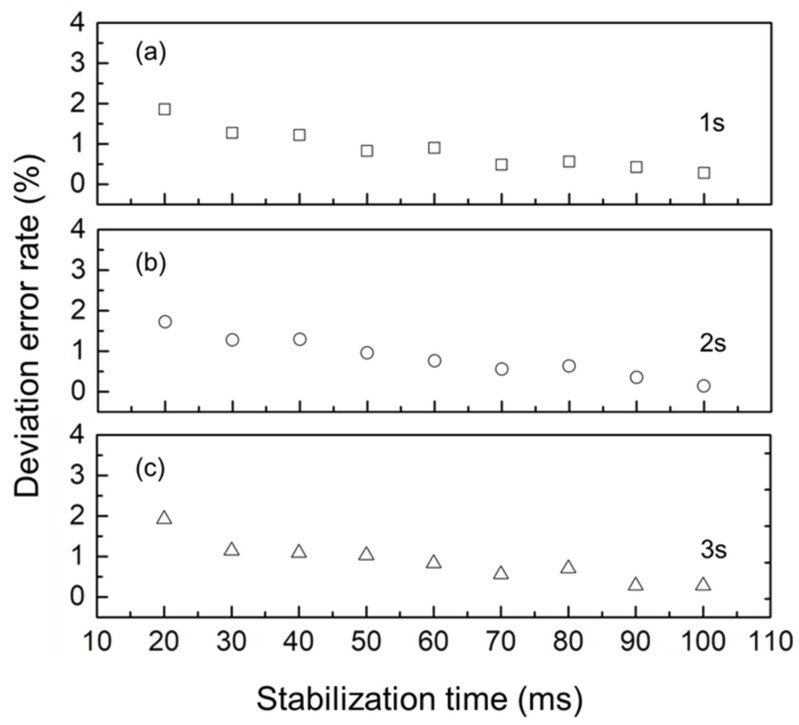
Comparison between the deviation error ratio and stabilization time. (**a**–**c**) Measurement interval time settings with 1, 2, and 3 s.

In a previous study, based on the preliminary evaluation of the sensor electrode and the simultaneous detection of only two channels, we set each sensor electrode with a specific measurement circuit, adopting a parallel detection method [16]. However, the new sensor electrode pattern can realize a 4 × 4 array, i.e., 16 channels are connected to the measurement system. The parallel detection method causes the measurement system to be too bloated; therefore, we used the series detection method (i.e., the same measurement circuit can correspond to the sensor electrode on the 16 channels). Using the series inspection method, the efficiency of the measurement circuit and power consumption can be greatly improved; however, the measurement circuit in the sensor system requires stabilization time during each channel switch to achieve accurate testing. Therefore, for the new series detection method, the stability characteristics of the sensor system must be tested. Figure 8 illustrates the relationship between the measurement deviation error ratio and the stabilization time of the sensor system. Specifically, Figure 8a–c correspond to the measurement interval time settings of 1, 2, and 3 s, respectively. Given that the primary purpose of this sensor is to test the urination behavior of animals, the frequency of a 3 s measurement interval time is sufficient to meet all requirements of use.

### 5.2. Simultaneous Field Measurement of Multiple Targets

In a previous study, we conducted a preliminary experiment to monitor the urination behavior of a single rat [16]. Owing to experimental limitations, only two sensor electrodes were printed in the central area of the mat. Consequently, the previous version of the sensor system could not provide a comprehensive quantitative analysis of urination monitoring. The current animal experiment serves as a supplementary study to previous field measurements, allowing for a more comprehensive evaluation of rat urination behavior. This supplementary work focuses on two main aspects. First, the electrode pattern design was optimized to better cover the active area of the rats. Second, in this experiment, 2 × 2 and 4 × 4 sensor electrode arrays were used to enhance the test accuracy and spatial resolution. During the experiments, the sensor system did not alter the living environment or the habits of the animals.

**Figure 9:**
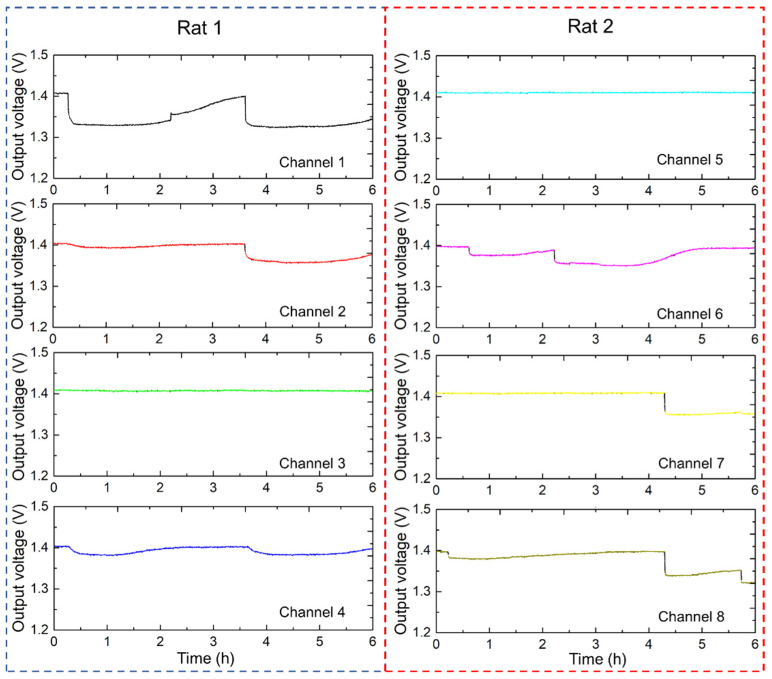
Results of the simultaneous urine monitoring experiment for multiple rats.

Figure 9 shows the continuous urination monitoring results for the two rats using the multi-channel sensor electrodes. The four-channel 2 × 2 sensor array system effectively captured and recorded urination patterns over a 6 h period at 1 min intervals. Each 2 × 2 sensor array, with a sensing area of 100 × 100 mm^2^, continuously recorded urination events. Sensor arrays were strategically placed to ensure comprehensive coverage of the residential areas of the rats. Channels 1 to 4 demonstrate the output voltage graphs for rat 1, indicating the occurrence and distribution of urination events. Periodic changes in output voltage signify multiple urination episodes. The intensity and frequency of these changes provide insights into urination behavior. Channels 5 to 8 displayed similar output voltage results for rat 2, with spikes indicating urination events. Differences in spike patterns between channels suggested variations in urination frequency and volume between the two rats. Use of the electrode array allowed the differentiation of urination events in different areas, which can be correlated with the activity and movement of rats within the monitored space. The temporal and spatial resolution of the data were sufficient to capture detailed urination patterns, which are crucial for studies on urinary health and behavior. Analyzing the differences in urination patterns between the two rat models can help identify any irregularities or specific behaviors.

### 5.3. Field Measurement of a Single Target with High Precision

**Figure 10:**
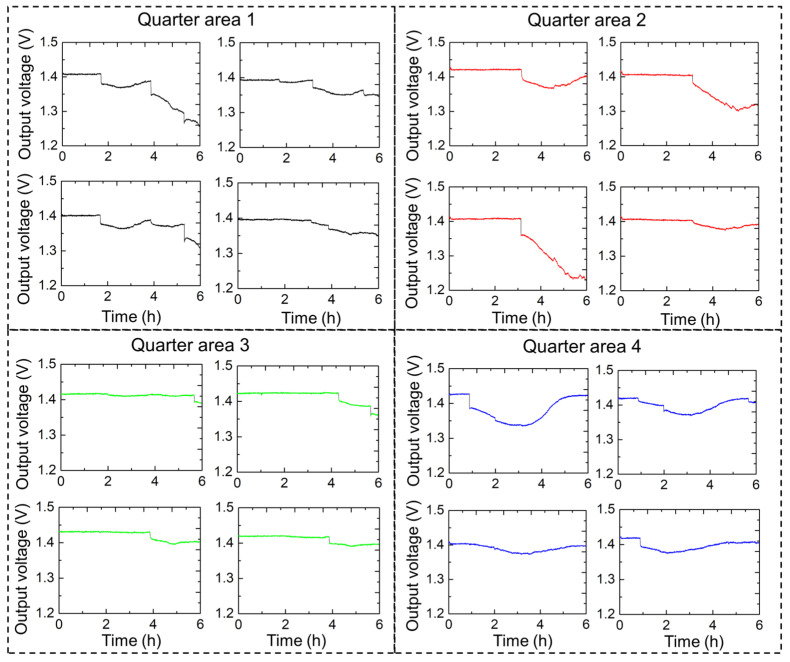
Sixteen-channel sensor system for urine measurement with high precision.

Subsequently, we sought to continuously monitor the urination patterns of a single rat over a 6 h period at 1 min intervals using a 4 × 4 sensor array. Figure 10 shows the continuous urination monitoring results for one rat with high precision. In this experiment, a 16-channel sensor array with a sensing area of 176 × 162 mm^2^ was used. The 4 × 4 sensor array was designed to provide high-resolution temporal and spatial data on urination events, thereby facilitating an understanding of urinary behaviors and potential abnormalities. The results are presented in a series of output voltage graphs, each corresponding to a quarter of the sensor array area. Quarters 1 and 2 showed periodic spikes in the output voltage, indicating multiple urination events. Significant peaks were observed at regular intervals, demonstrating the urination frequency of the rat within these regions. Quarter 3 indicated fewer but more pronounced urination events, possibly reflecting the movement and preference of the rat for specific urination regions. Quarter 4 also showed distinct changes in output voltage, although the pattern may differ from that of the other quarters, providing a comprehensive overview of the spatial distribution of urination.

Data recorded from the 16-channel diaper sensor provided valuable insights into the urination behavior of rats. Several parameters can be evaluated by analyzing the output voltage spikes, including the frequency of urination, duration of urination events, and volume of urination. A continuous monitoring system using the 16-channel diaper sensor effectively captured detailed urination patterns, which are crucial for studies related to urinary health and behavior in rats. The spatial distribution of urination events across different quarters can help identify preferences and potential issues in urinary function. Moreover, these data can be used to correlate urination patterns with other physiological and behavioral parameters, contributing to a holistic understanding of rat health. Thus, the 16-channel diaper sensor provides a reliable and detailed method for monitoring urination in rats. The high-resolution data obtained in this study can enhance our understanding of urinary behavior and identify potential health issues, making it a valuable tool in biomedical research. The recorded data enabled the identification of patterns, such as regularity or irregularity, in urination intervals. Consistent intervals may indicate normal physiological functions, whereas irregular intervals may indicate underlying health issues. Regular intervals between urination events suggest a stable physiological state and proper functioning of the urinary system, whereas irregular intervals could indicate potential issues, such as urinary tract infections, bladder dysfunction, or other health concerns that may require further investigation.

## 6. Discussion and Summary

Functional improvements to the sensor system should be considered in future field measurements and commercial applications. Printed thin sensor electrodes exhibit inherent material characteristics, resulting in relatively high sheet resistance, which affects the measurement accuracy and complicates signal detection. To address these issues, materials with better conductivities should be selected for printing. This choice will reduce the sheet resistance of the sensor electrodes, enabling the use of thinner and longer electrode beams and simpler detection circuits. The sensor electrodes are directly printed on photographic paper, a production method that significantly reduces costs. However, because electrodes are exposed to air, they are prone to scratching and damage, which reduces their service life. For long-term use, protective coating can be applied to the printed electrodes to extend their durability. This study presents a highly efficient and cost-effective solution for real-time urine monitoring, outperforming existing devices. The proposed sensor offers a high level of user adaptability, allowing for the rapid customization of sensor electrode configurations according to different testing requirements, thereby considerably reducing preparation time.

To facilitate data organization, the output values of the sensor system were recorded as the original voltage data. In field experiments, the relationship between the sensor output voltage and urine volume can be established through calibration, which allows users to easily determine the urine volume. A tablet is used as the terminal monitor and controller of the communication system, which is convenient owing to its large screen size. For applications requiring improved mobility and compactness, a smartphone-based terminal client can be provided to meet different needs. Future work will focus on further optimizing the sensor design to enhance performance and explore additional applications in health monitoring. The ultimate goal is to contribute to the well-being of the older population through innovative diagnostic and treatment devices that offer reliable and effective monitoring solutions.

In summary, this study introduces a urinary sensor array with extended electrodes created using rapid printing technology to achieve the precise measurement of urine volume and frequency. The ultrathin design of the sensor electrode, with a thickness of only a few tens of microns, ensures both user comfort and measurement accuracy. The dimensions of the sensor electrode were designed, measured, and optimized. The trial manufacturing of the sensor electrode and array was successfully implemented. We conducted a comprehensive evaluation of the fabricated sensor electrodes, including tests for response speed, interval performance, and long-term stability. In addition, we developed wireless transmission capabilities and user interfaces for field experiments. The final stage of our study involved animal measurements to evaluate the field performance of the fabricated sensors. In conclusion, the developed urinary sensor array demonstrates remarkable potential for improving health monitoring. Its precise measurement, user comfort, and robust performance make it a valuable tool for advancing healthcare technology. Future work will aim to further optimize the sensor design and expand its applications in various health monitoring scenarios, ultimately contributing to the well-being of the aging population through innovative diagnostic and treatment devices. Potential applications include monitoring urination in newborns and the real-time tracking of urination patterns in older individuals, particularly those requiring care. We are also actively collaborating with medical care institutions, aiming to apply the sensor to human health detection in future studies.

## Data Availability

All data are true and reliable.
